# Clinicopathological analysis of ovarian sertoli-leydig cell tumor with postmenopausal vaginal bleeding as the first symptom: A case report

**DOI:** 10.1097/MD.0000000000024922

**Published:** 2021-04-02

**Authors:** Li-juan Huang, Liang-yan Shi, Jie Duan

**Affiliations:** a Medical School, Wuhan University of Science and Technology; b Department of Obsterics and Gynecology, Hubei Maternal and Child Health Hospital Affiliated to Huazhong University of Science and Technology, Wuhan, China.

**Keywords:** ovarian sertoli-leydig cell tumor, postmenopausal vaginal bleeding, testosterone

## Abstract

**Rational:**

Ovarian sertoli-leydig cell tumor (OSLCT) is extremely rare. We reported a OSLCT case in whom postmenopausal vaginal bleeding was the first symptom.

**Patient concerns:**

The patient came to our hospital due to postmenopausal vaginal bleeding.

**Diagnoses:**

Serum tumor markers and color Doppler ultrasound for her pelvic cavity were negative. The patient was finally diagnosed with left OSLCT by pathology. It was difficult to make a definite diagnosis before operation, the diagnosis of OSLCT required postoperative pathology in the patients.

**Interventions:**

the patient underwent laparoscopic hysterectomy+bilateral adnexectomy+lysis of pelvic adhesions.

**Outcomes:**

Postoperative laboratory examinations were normal. The patient was discharged from our hospital on the seventh day after operation and came to our hospital for follow-up check in April 2020. Physical and laboratory examinations were normal.

**Lessons:**

OSLCT can show different endocrine abnormalities, which are related to the various types of tumor tissues. Missed diagnosis and misdiagnosis are likely to occur in the patients who only have elevated serum testosterone. For the menopausal women with elevated serum testosterone, ovarian tumor shoule be highly suspected after excluding adrenal gland-related diseases.

## 1 Introduction

Ovarian sertoli-leydig cell tumor (OSLCT), a subtype of ovarian sex cord stromal cell tumor, is extremely rare and accounts for less than 0.5% of ovarian tumors.^[1]^ OSLCT is usually unilateral and occurs in young women.^[2]^ It is a functional ovarian tumor, with androgen secretion, leading to virilization and de-feminization.^[3]^ OSLCT is mainly diagnosed by postoperative biopsy because preoperative clinical features are not marked in most OSLCT patients. According to different tissue morphologies, OSLCT is divided into high, middle, and low differentiation. At present, high-differentiated OSLCT is regarded as benign, while middle or low-differentiated OSLCT containing reticular growth pattern or heterogenic component is thought to have malignant biological behaviors.^[4,5]^ There are a few reports on OSLCT due to its low incidence. In order to provide a reference for diagnosis and treatment of OSLCT, we reported a patient in whom OSLCT was incidentally found during laparoscopic hysterectomy plus bilateral adnexectomy performed for postmenopausal irregular vaginal bleeding.

## 2 Case report

All study methods were approved by Institutional Review Board and Ethics Committee of Hubei Maternal and Child Health Hospital. The patient reported in this study gave written inform consent to publish the case details and images.

A 61-year-old woman, who attained menopause when she was 50 years old, was referred to our hospital on July 8th, 2019 due to 2-year intermittent vaginal bleeding. She complained of intermittent vaginal bleeding since 2017. A diagnostic curettage was performed in a local hospital. The patient said that she did not receive any further treatment in the local hospital because the diagnostic curettage displayed benign lesions. In February and October 2018, as well as June 2019, new episodes of vaginal bleeding occurred and then, the patient was referred to our service. The patient had a history of 3-year hypertension (10 mg of oral extended release nifedipine tablets, once a day), but she had no history of diabetes. Her body mass index (BMI) was 27.6 kg/m^2^.

Physical examination showed the following: vulva inspection was unremarkable. A small amount of blood was collected in the vagina. A tumor of 3 mm × 3 mm was observed in the uterine cervix. The uterus was slightly enlarged with mobility and adnexa were not palpable.

Gynecological color Doppler transvaginal ultrasound showed enlarged uterus (6.7 cm × 5.8 cm × 5.0 cm), endometrial thickness of 1.6 cm, nabothian cysts and normal bilateral appendages (the left ovary of 2.4 cm × 2.0 cm × 1.5 cm and the right ovary of 2.1 cm × 1.5 cm × 0.6 cm).

Laboratory examination was as follows: results of thyroid function tests and the level of human chorionic gonadotrophin (β-HCG) were normal. Tumor markers were negative. The preoperative abnormal results of laboratory examinations and their reference values for menopause are shown in Table 1.

**Table 1:** Preoperative levels of other laboratory examinations and their reference values for menopause.

Items	FSH (mIU/mL)	LH (mIU/mL)	Estradiol (pg/mL)	P (ng/mL)	L (ng/mL)	T (ng/mL)	BFS (mmol/L)	2-h BS (mmol/L)
Levels	10.77	6.94	48.27	0.130	24.5	0.720	8.66	7.97
RV	25.8–134.8	7.7–58.5	<49.9	<0.05–0.126	4.79–23.3	0.029–0.408	3.9–6.1	3.9–7.8

BFS = blood-fasting sugar, BS = blood sugar, FSH = follicle stimulating hormone, L = lactotropin, LH = luteinizing hormone, P = progesterone, RV = reference values, T = testosterone.

A hysteroscopy followed by uterine curettage was performed on July 10th, 2019. The cervical lesion and an endometrial polyp were excised. The histopathological results were as follows: endometrial polyp with endometrial typical hyperplasia, and cervical polyp. In the days following the procedure, several new episodes of vaginal bleeding occurred. A laparoscopic hysterectomy with bilateral adnexectomy and lysis of pelvic adhesions was performed on July 17th, 2019.

Intraoperative findings were as follows: uterine size of 7.0 cm × 7.0 cm × 6.0 cm with smooth surface, left ovary 3.0 cm × 2.0 cm × 1.0 cm with upper part showing dark red granular aspect. Adhesion of small intestine with the proper ligament, left fallopian tube without macroscopic peculiarities was observed. Right ovary of size 3.0 cm × 2.0 cm × 1.0 cm and right fallopian tube without macroscopic peculiarities was observed. The intraoperative frozen pathology suggested possibility of left ovarian sex cord stromal tumor.

Gross appearance was as follows: the left ovary of size 2.5 cm × 2 cm × 1.5 cm with gray yellow and gray white sections, and the right ovary of size 2 cm × 1.5 cm × 0.5 cm with gray white sections.

**Figure 1:**
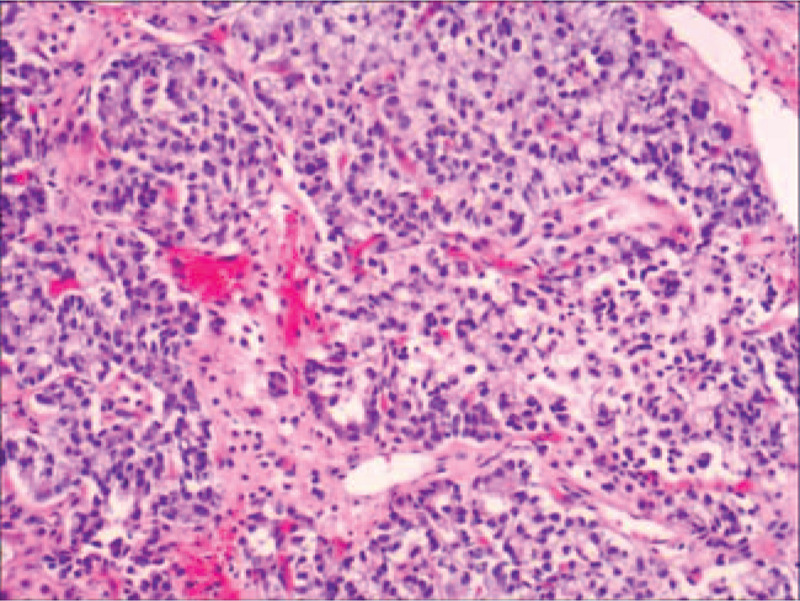
Ovarian sertoli-leydig cell tumor under microscope, nodular structures are divided into lobes by fibrous bands, and consist of hollow tubules and solid tubules. In tubular lining, there are sertoli cells which are columnar, round, or oval without obvious nuclear atypia and karyokinesis. In interstitium, there are clusters of highly differentiated leydig's cells containing lipids and less Reinke crystallization. Based on above findings, it is consistent with middle-differentiated OSLCT. Reticular growth pattern and heterogenic component are not found in the ovarian lesion, and tumor embolus is also not seen in vessels. OSLCT = ovarian sertoli-leydig cell tumor.

Pathological diagnosis: under microscope for the left ovary, nodular structures were divided into lobes by fibrous bands, and consisted of hollow tubules and solid tubules. In tubular lining, there were sertoli cells which were columnar, round, or oval without obvious nuclear atypia and karyokinesis. In interstitium, there were clusters of highly differentiated leydig's cells containing lipids and less Reinke crystallization. Based on above findings, it was consistent with middle-differentiated OSLCT. Reticular growth pattern and heterogenic component were not found in the left ovarian lesion, and tumor embolus was also not seen in vessels (Fig. 1). Immunohistochemical results were EMA(−), PCK(+), ER(−), PR(spot focus +), P53(part +), CK7(−), CK8(+), CK20(−), Calretinin(part +), Inhibin-α(+), SALL4(−), Syn(spot focus +), CD56(+), CgA(−), CD99(+), Melan-A(+), WT1(−), Ki67(Li: 7%), PAS(−).

Postoperative laboratory examinations indicated progesterone <0.050 ng/mL (normal range for menopause <0.05–0.126), testosterone <0.030 ng/mL (normal range for menopause: 0.029–0.408), lactotropin of 18.17 ng/mL (normal range for non-pregnant women: 4.79–23.3), estradiol of 6.64 pg/mL (normal range for menopause <49.9), luteinizing hormone of 13.60 mIU/mL (normal range for menopause: 7.7–58.5), and follicle stimulating hormone (FSH) of 28.80 mIU/mL (normal range for menopause: 25.8–134.8) on the fifth day after operation (July 22, 2019). Due to the favorable histopathological characteristics, no adjuvant therapy was performed. The patient was discharged from our hospital on the seventh day after operation (July 24, 2019) and came to our hospital for follow-up check in April 2020 (the time period of follow-up in 9 months). Her blood pressure was controlled within normal range (10 mg of oral extended release nifedipine tablets, once a day) and other physical examinations were normal. Her fasting blood glucose was controlled between 5.1 and 6.7 mmol/L, and her highest postprandial blood glucose was less than 14 mmol/L under oral metformin twice a day.

## 3 Discussion

OSLCT is very rare and its pathogenesis is still unclear. It has been reported that some genes such as DICER1, STK11, and FOXL2, or somatic mutations are associated with ovarian sex cord stromal tumor.^[6]^ In the patient of this study, gene analysis was not performed, so it was not known whether there was abnormal gene expression.

### 3.1 Clinical features

OSLCT is a rare female reproductive tumor and accounts for less than 0.5% of ovarian tumors. OSLCT may occur at any age from 6 months to 75 years old, but most occur at 25 years of age and less than 10% of OSLCT occur before menarche or after menopause.^[1]^ OSLCT consisted of sertoli cells, stromal cells, and fibroblasts in different proportions and different differentiating degree. Sertoli cells mainly produce estrogen and a little of progestogen and androgen, while leydig's cells mainly produce androgen and a little of progestogen and estrogen.^[7]^ Therefore, the clinical manifestations of OSLCT vary according to different compositions of OSLCT. A typical clinical feature is de-feminization and masculinization, suggesting that early action of androgen is mainly characterized by de-feminization such as infrequent menstruation or amenorrhea, infertility, and breast atrophy followed by masculinization such as hirsutism, clitorimegaly, and occurrence of Adam's apple. The action of estrogen is mainly characterized by female pseudoprecocious puberty, menorrhagia, menstrual disorder, or postmenopausal vaginal bleeding. Some OSLCT patients also present with abdominal pain, abdominal distension, or abdominal mass, but have no endocrine disorder-related symptoms. In this study, the patient was a 61-year-old woman with postmenopausal vaginal bleeding as the first symptom, and she also had adenomyosis, uterine adenomyoma, endometrial irregular hyperplasia, and endometrial polyps, which suggested that estrogen played a leading role in her body. However, in the patient, estrogen level was near to its normal upper limit; while testosterone level was over normal upper limit. This patient did not develop androgen-related clinical manifestations, probably because of an overexpression of aromatase P450 (CYP19A1) that converted androgen into estrogen.^[8]^ After operation, the estrogen level dropped sharply from 48.27 pg/mL to 6.64 pg/mL, and testosterone level from 0.720 ng/mL to 0.030 ng/mL in the patient.

Functional ovarian tumors are commonly ovarian sex cord stromal tumors including steroid cell tumor, leydig cell tumor, granulosa cell tumor, sertoli-cell tumor, sertoli-leydig cell tumor, gonadoblastoma, and other rare metastatic ovarian tumors from endocrine tumors. In this patient, only testosterone level was over normal upper limit, but it did not reach its warning level of 6 nmol/L,^[9]^ so it was difficult to diagnose ovarian tumor before operation.

Some patients with OSLCT may have metabolic diseases such as hypertension and diabetes. It was reported that a 56-year-old patient who had postmenopausal vaginal bleeding and was diagnosed with OSLCT, had a history of hypertension and type 2 diabetes.^[7]^ Metabolic diseases such as hypertension and diabetes are common in the elderly, so it is not often considered that they are caused by ovarian tumor. Whether metabolic diseases are related to OSLCT remains to be further confirmed. It has been reported that in postmenopausal women, the elevated androgen level increases the risks for cardiovascular disease and insulin resistance,^[10]^ and plays a key role in metabolic syndrome.^[11]^ In this patient, her hormone level returned to normal after operation. It is difficult to confirm that whether her hypertension and diabetes were associated with OSLCT.

### 3.2 Histopathological features

OSLCTs are usually confined to unilateral ovary, and only less than 3% of the OSLCTs spread throughout the whole ovary.^[2]^ Most OSLCTs are classified as stage I and its diagnosis is mainly based on histopathology. OSLCTs are generally cystic solid mass with gray red or grayish brown sections, bleeding, and necrosis. Under microscope, OSLCT consists of sertoli cells and leydig's cells in different proportions. The sertoli cells are arranged into tubular structures, and the leydig's cells are distributed around them. According to the different tissue morphologies, OSLCT is divided into high, middle, and low differentiation, as well as reticular growth pattern and heterogenic component.^[5]^ It was reported that a middle-differentiated OSLCT was cystic and solid mass with smooth surface, and there was transparent yellow liquid in the cyst and papilla on the cystic wall.^[4]^ It is generally believed that the smaller tumor has better differentiation.^[12]^ For the OSLCT with atypical clinical and pathological features, immunohistochemistry is necessary. In OSLCT, sertoli cells usually express calretinin, inhibin-α, keratin, vimentin, and CD99, while leydig's cells express vimentin, inhibin-α, calretinin, and Mela-A.^[13]^ In this study, inhibin-α, CD99, calretinin, and Mela-A were positive. The specificity of inhibin-α is strong, and the sensitivity of calretinin is high, so inhibin-α and calretinin are usually applied together in diagnosis of OSLCT.^[14]^ However, OSLCT has no absolutely specific immunohistochemical markers, so immunohistochemical results only are used as a reference in diagnosis of OSLCT.

### 3.3 Treatment for OSLCT

At present, there is no standard treatment for OSLCT, surgery is still the first treatment option. The choice of operation method is based on age, fertility requirement, clinical stage, tumor size, and differentiation degree. High risk factors for SLCTs include advanced clinical stage, low differentiation, large tumor, and presence of reticular growth pattern or/and heterogenic component. For the patients who have high-differentiated OSLCT but do not require to preserve fertility, the affected ovary combined with affected-sided appendage or double-sided appendages are usually removed, or hysterectomy combined with bilateral adnexectomy is performed. The OSLCT patients with high risk factors often receive comprehensive staging operation, namely that hysterectomy + bilateral adnexectomy + appendectomy + pelvic lymphadenectomy.^[15]^ However, Gui et al^[16]^ believed that pelvic plus aortocaval lymphadenectomy was not necessary due to rare lymphatic metastasis in OSLCT. According to the clinical stages and pathological results made by Federation International of Gynecology and Obstetrics (FIGO),^[17]^ the patient in this study was diagnosed with stage 1A OSLCT (middle-differentiation). In the patient reported here, she was 61-year-old and menopausal and she did not have OSLCT-related high risk factors; so only hysterectomy plus bilateral adnexectomy were performed in this patient.

Chemotherapy is usually used in the OSLCT that has advanced clinical stage, middle or low-differentiation, active karyokinesis or/and heterogenic component; and the chemotherapy protocol (bleomycin, etoposide, and cisplatin) is often applied in OSLCT.^[18]^ It is reported that clinical staging is an important indicator for OSLCT postoperative chemotherapy.^[19]^ The 5-year survival rate is about 95% for stage I patients and zero for stage III or IV patients.^[1]^ The patient in this study did not receive chemotherapy and no recurrence was found until the last follow-up (April 18, 2020).

## 4 Conclusion

Ovarian tumors combined with endocrine changes often suggest the possibility of ovarian tumors. However, the preoperative diagnosis of OSLCT is difficult when OSLCT clinical manifestations are not obvious and the ovarian tumor is too small to be detected by imaging examinations. This study suggests that for the menopausal women with irregular vaginal bleeding, abnormal endocrine test, history of hypertension or/and diabetes, ovarian endocrine-related tumors should be highly suspected for OSCLT after excluding endometrial lesions and related endocrine diseases.

## Author contributions

**Conceptualization:** Jie Duan.

**Data curation:** Li-juan Huang, Liang-yan Shi.

**Writing – original draft:** Li-juan Huang.

**Writing – review & editing:** Jie Duan.
